# Chronic DON exposure and acute LPS challenge: effects on porcine liver morphology and function

**DOI:** 10.1007/s12550-017-0279-9

**Published:** 2017-05-04

**Authors:** Lydia Renner, Stefan Kahlert, Tanja Tesch, Erik Bannert, Jana Frahm, Anikó Barta-Böszörményi, Jeannette Kluess, Susanne Kersten, Peter Schönfeld, Hermann-Josef Rothkötter, Sven Dänicke

**Affiliations:** 1 Institute of Anatomy, Otto von Guericke University Magdeburg, Leipziger Str. 44, 39120 Magdeburg, Germany; 2 Institute of Animal Nutrition, Friedrich-Loeffler-Institute, Federal Research Institute for Animal Health, Bundesallee 50, 38116 Braunschweig, Germany; 3 Institute of Biochemistry and Cell Biology, Otto von Guericke University Magdeburg, Leipziger Str. 44, 39120 Magdeburg, Germany

**Keywords:** Deoxynivalenol, Lipopolysaccharide, Pig, Liver, Mitochondria

## Abstract

The aim of the present study was to examine the role of chronic deoxynivalenol (DON) exposition on the liver morphology and function in combination with pre- and post-hepatic lipopolysaccharide (LPS) stress in young pigs fed for 4 weeks with a DON-contaminated diet (4.59 mg/kg feed). At the end of the experiment, LPS (7.5 μg/kg BW) was administered for 1 h pre-hepatically (*Vena portae hepatis*) or post-hepatically (*Vena jugularis*). Liver morphology was macroscopically checked and showed haemorrhage in all LPS groups, significantly higher relative liver weights, accompanied by marked oedema in the gallbladder wall. Histological changes were judged by a modified histology activity index (HAI). Liver HAI score was significantly increased in all LPS groups compared to placebo, primarily due to neutrophil infiltration and haemorrhage. DON feed alone was without effect on the liver HAI. Liver function was characterized by (i) hepatic biochemical markers, (ii) mitochondrial respiration and (iii) Ca^2+^ accumulation capacity of isolated mitochondria. Clinical chemical parameters characterizing liver function were initially (<3 h) slightly influenced by LPS. After 3 h, bilirubin and alkaline phosphatase were increased significantly, in DON-fed, jugular-infused LPS group. Respiration and Ca^2+^ accumulation capacity of isolated liver mitochondria was not impaired by chronic DON exposure, acute LPS challenge or combined treatments. DON-contaminated feed did not change macroscopy and histology of the liver, but modified the function under LPS stress. The different function was not linked to modifications of liver mitochondria.

**Electronic supplementary material:**

The online version of this article (doi:10.1007/s12550-017-0279-9) contains supplementary material, which is available to authorized users.

## Introduction

Contamination of crops with the *Fusarium* toxin deoxynivalenol (DON) is frequently observed in Central Europe (EFSA 2013), and swine as the most sensitive species shows alterations of the immune system and protein synthesis after DON exposure, but often with contradictory results (Rotter et al. 1996; Kullik et al. 2013), dependent on toxin dose and exposure time. In several studies, trichothecenes affected also cellular and mitochondrial properties (Pace 1983), for example diminishing oxygen consumption of primary cardiomyocytes exposed to low-dose DON (Ngampongsa et al. 2013) or in higher doses (100 μM DON), triggering cell apoptosis by the mitochondria-associated pathway (Bensassi et al. 2012). In contrast to such acute, direct mitotoxic effects, it has been shown that an application of low-dose DON (200 ng/mL corresponding to 0.68 μM) to the basolateral compartment of membrane cultured IPEC-J2 cells triggered the messenger RNA (mRNA) expression of components of the citrate cycle and the oxidative phosphorylation (Diesing et al. 2012), indicating the crucial role of mitochondria. Harmful reactive oxygen species (ROS) are generated by mitochondrial respiration, leading potentially to oxidative stress. In a recent review, more than 20 in vitro studies demonstrated DON’s involvement in an oxidative stress response, but less than ten studies dealt with this problem in vivo (Mishra et al. 2014).

Lipopolysaccharides (LPS) are located in the outer membrane of gram-negative bacteria, acting as endotoxins (Cohen 2002), and this mechanism has been widely used in experimental models inducing immunomodulation with low endotoxin levels and septic models with high LPS doses (Wyns et al. 2015). Previously, it has been demonstrated that pigs showed an attenuated immune response after challenge with ovalbumin (Grenier et al. 2011) or sheep red blood cells (Rotter et al. 1994) when previously exposed to dietary *Fusarium* toxins. Such a “priming effect” of DON was also demonstrated in a porcine endotoxaemic model (Stanek et al. 2012), where dietary DON exposure also partially attenuated LPS induced hepatic lesions when compared to control-fed counterparts.

Both, deoxynivalenol and LPS are primarily detoxified in the liver and we reported earlier that a post-hepatically induced acute phase reaction (APR) differed from a pre-hepatic provoked one (Bannert et al. 2015; Tesch et al. 2015). We thus hypothesize that the functional hepatic capacity can be modified by a chronic dietary DON burden, and this priming may result in an attenuated response of liver mitochondria and function to an immune challenge in vivo, dependent on its route of administration. In order to clarify this hypothesis, we used specimens collected from the above cited experiment (Bannert et al. 2015; Tesch et al. 2015).

## Materials and methods

### Animal experiment

**Fig. 1:**
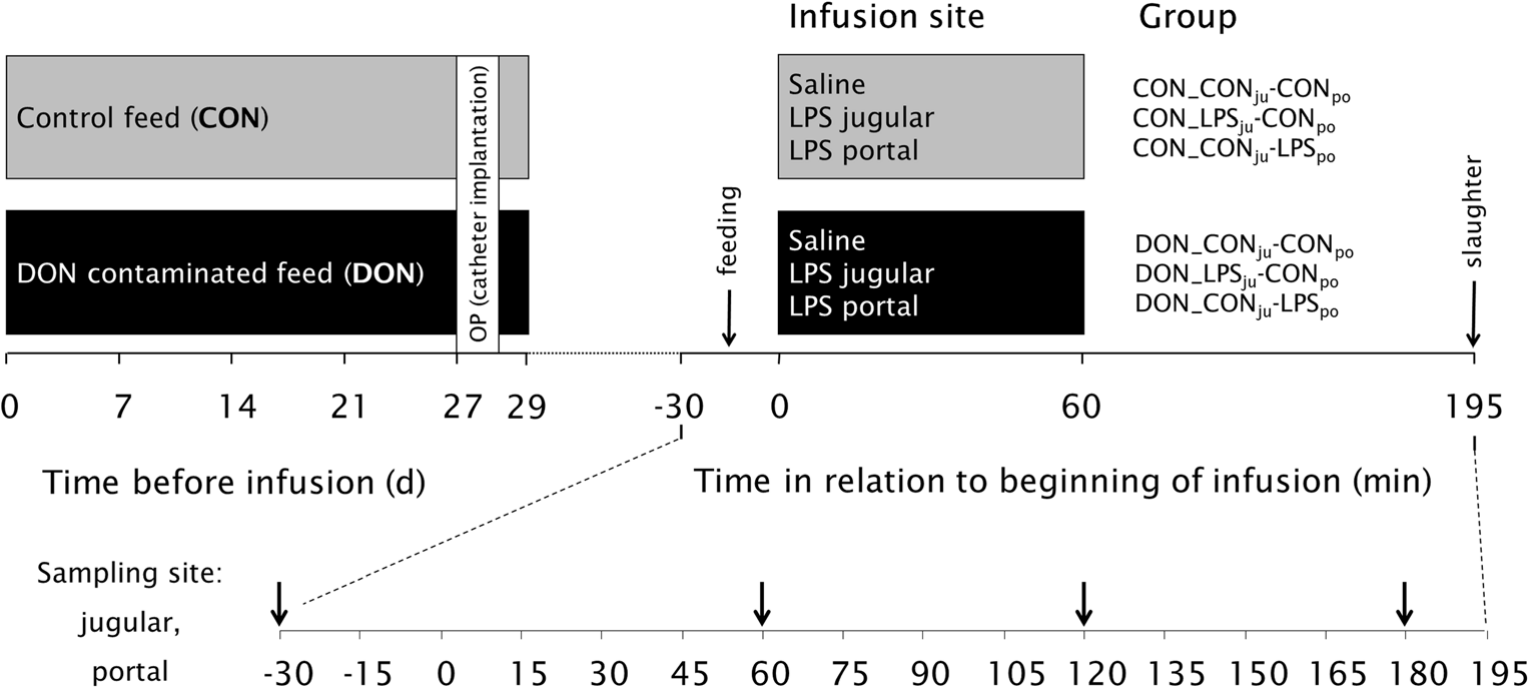
Design of the animal experiment. Barrows (*n* = 42) received either a control feed (*CON*) or a DON-contaminated feed (*DON*; 4.59 mg/kg feed) for 4 weeks; at the end of the experiment, *E. coli* LPS (7.5 μg/kg BW) or 0.9% saline was infused into jugular (*ju*) or portal (*po*) region

The animal trial was performed in the Friedrich-Loeffler-Institute (Braunschweig, Germany) and approved by the ethical committee of the Lower Saxony State Office for Consumer Protection and Food Safety (file number 33.4-42502-04-13/1274) and conducted according to the European Community regulations concerning the protection of experimental animals and the guidelines of the German Animal Welfare Act. This trial is part of a large project and data on animal health and physiology are already published elsewhere (Bannert et al. 2015; Tesch et al. 2015, 2017). In brief, 42 barrows (German Landrace, Mariensee, Germany) with an initial body weight of 25.8 ± 3.7 kg were divided equally in two dietary groups, receiving either a control or a DON-contaminated diet (4.59 mg/kg feed) for 4 weeks. Pigs were fed 700 g (air-dry matter, ADM) twice daily, provided as slurry. The main components of the diet (Tesch et al. 2015) were barley (533 g/kg dry matter, DM), maize (150 g/kg DM, where 75 g/kg were replaced by DON-contaminated maize for DON groups), soybean meal (200 g/kg DM), rapeseed (50 g/kg DM) and soybean oil (20 g/kg DM). At day 27 of the experiment, pigs were surgically equipped with post-hepatic catheters in *Vena jugularis interna et externa* and pre-hepatically in *Vena lienalis* and *Vena portae hepatis* in order to facilitate simultaneous infusion and blood sampling. At day 29, 15 min after morning feeding, LPS (7.5 μg/kg BW dissolved in 0.9% saline, *Escherichia coli* O111:B4, Product number L2630, Sigma-Aldrich, St. Lois, MO, USA) or saline (CON) was infused via *V. jugularis externa* (post-hepatic administration) and *V. lienalis* (pre-hepatic administration), respectively, for 1 h. Thus, two dietary groups (CON vs. DON) and three infusion regimens (NaCl, LPS_portal_, LPS_jugular_) resulted in six experimental groups, whereby the first abbreviation denotes diet and the second the infusion regimen: CON_CON_ju_-CON_po_, CON_LPS_ju_-CON_po_, CON_CON_ju_-LPS_po_, DON_CON_ju_-CON_po_, DON_LPS_ju_-CON_po_ and DON_CON_ju_-LPS_po_ (Fig. 1). Pigs were sacrificed 195 min after start of infusion, and the liver and the gallbladder were immediately removed from the abdominal cavity for further analyses.

### Macroscopic and microscopic inspection

The liver and the gallbladder from sacrificed animals were removed and photographed. The liver was weighed and subsequently samples were taken for liver mitochondria extraction and for histopathological examination. Samples for histopathology were fixed in 4% formaldehyde (Histofix, Roth GmbH, Germany). These samples were dehydrated in increasing ethanol concentrations and embedded in paraffin. Sections (3–5 μm) were cut on a HM 355S rotation microtom (Microm International GmbH, Germany) and mounted on glass slides (2 sections/slide). Specimens were HE stained and blinded sections (2 sections/sample) were evaluated (brightfield microscopy) using the modified histology activity index (HAI) based on Ishak et al. (1995) and modified by Stanek et al. (2012). The modified HAI incorporates signs of inflammation (portal, periportal and acinar infiltration of neutrophil and eosinophil granulocytes), necrosis (focal and confluent) and haemorrhages (Fig. S1). All parameters were summed up for a cumulative HAI with a possible maximum score of 40, representing the highest damage evaluated.

Exemplary formaldehyde-fixed samples of gallbladder wall were paraffin-embedded; sections (3–5 μm) were cut on a HM 355S rotation microtom (Microm International GmbH, Germany) and mounted on glass slides. Specimens were stained with a Masson-Goldner trichrome staining.

### Clinical chemical parameters

Blood was taken via the portal and jugular catheter 30 min before LPS/saline infusion and 60, 120 and 180 min *post infusionem* (S-Monovette®, Sarstedt). Whole blood samples were left clotting for 60 min at room temperature and then for another 30 min at 30 °C. Samples were centrifuged at 2123×*g* for 15 min (15 °C), and serum was stored at −80 °C until further processing. The following clinical chemical parameters were measured on an automated analyser (Eurolyser CCA180, Eurolab, Austria): albumin (ALB), total protein, glutamate dehydrogenase (GLDH), aspartate aminotransferase (AST), gamma-glutamyltransferase (γGT), alkaline phosphatase (ALP) and total bilirubin.

### Mitochondrial function

Liver samples of similar weight were taken from the left hepatic lobe, and isolation of pig mitochondria was done by differential centrifugation as described in Steinbrecht and Kunz (1970). Briefly, the removed liver pieces were cleaned from vessels and, thereafter, cut into smaller pieces with a scissor. All solutions applied were kept at 4 °C. After washing liver pieces with a solution of 0.25 M sucrose, pieces were transferred into medium composed of 0.25 M sucrose, EDTA (1 mM) and bovine serum albumin (0.1%), adjusted to pH 7.4 by Tris. Thereafter, liver pieces were homogenized using a Potter-Elvehjem homogenizer, and the obtained suspension was centrifuged for 5 min at 600×*g*. After filtration of the supernatant through gauze, the solution was centrifuged for 4 min at 5100×*g*, and the obtained pellet was re-suspended in 0.25 M sucrose/Tris with a smaller Potter-Elvehjem homogenizer. Next, the suspension was centrifuged for 2 min at 12300×*g*, and the obtained pellet again homogenized. Finally, after a further centrifugation for 10 min at 12300×*g*, the mitochondrial pellet was re-suspended in 0.25 M sucrose Ultra C/Tris. The protein content in the mitochondrial stock suspension was estimated by a modified biuret method. The biuret reagent was supplemented with 3% desoxycholate for solubilization of membrane proteins (Steinbrecht and Kunz 1970).

#### Oxygen consumption measurement

Incubation medium contained 110 mM mannitol, 60 mM KCl, 60 mM Tris-HCl, 10 mM KH_2_PO_4_ and 0.5 mM EGTA (pH 7.4). Oxygen consumption of mitochondria was measured using an Oxygraph® from Oroboros Instruments (Innsbruck, Austria) at 37 °C. For this purpose, an aliquot of mitochondrial suspension (0.75 mg protein/mL) was added to the incubation medium. Suspended mitochondria were energized by an addition of 5 mM glutamate plus 5 mM malate. Oxygen uptake was measured in the absence (state IV) and in the presence of 1 mM ADP (state III). Only if the measured respiratory control index (RCI; ratio between state III/state IV respiration) was higher than 3, the mitochondrial preparation was used for estimation of a possible effect of DON feed and LPS challenge on the respiration rate or the calcium retention capacity (CRC).

#### CRC

CRC was assessed fluorimetrically with 0.1 μM Calcium green-5 N (CaG5N) as membrane impermeable indicator for extramitochondrial Ca^2+^ (506 nm excitation and 532 nm emission wavelength) using a PerkinElmer Luminescence Spectrometer LS 50B (940 Winter Street, Waltham, MA 02451, USA). Briefly, aliquots of a 2-mM CaCl_2_ solution were added in a stepwise manner to incubations containing 1 mg of mitochondrial protein in a slightly modified incubation medium (1 mM KH_2_PO_4_, EDTA-free). Mitochondria were energized with 5 mM glutamate plus 5 mM malate as substrates. The uptake of added Ca^2+^ was followed as decrease of the CaG5N fluorescence. Measurements were done in presence and absence of cyclosporine A (CsA) as inhibitor of the mitochondrial permeability transition pore (PTP). Opening of the PTP is indicated by the sudden increase of Ca^2+^-CaG5N fluorescence.

### Statistical analyses

Clinical chemical parameters were analysed using the procedure “PROC MIXED” in SAS Enterprise Guide 6.1 (SAS Institute 2013, Cary, NC, USA) with −30 values as co-variable and group, sampling site, time and their interactions as fixed factors. A “REPEATED” statement was included for factor time accounting for the individual similarity at repeated measurements. Relative liver weight was also analysed by the same procedure, but only with group as fixed factor. Significant effects were further evaluated using the multiple *t* test (“pairwise differences,” PDIFF). Results are presented as least square means (LSMeans) and pooled standard error of means (PSEM).

Because HAI data represent a score and thus do not follow a Gaussian distribution, a non-parametric Kruskal-Wallis test with subsequent Dunn’s post-hoc test was performed using IBM SPSS Statistics (version 22). Data of mitochondrial function were evaluated by analysis of variance (ANOVA) using IBM SPSS Statistics (version 22).

## Results

### Macroscopy of liver and gallbladder

**Fig. 2:**
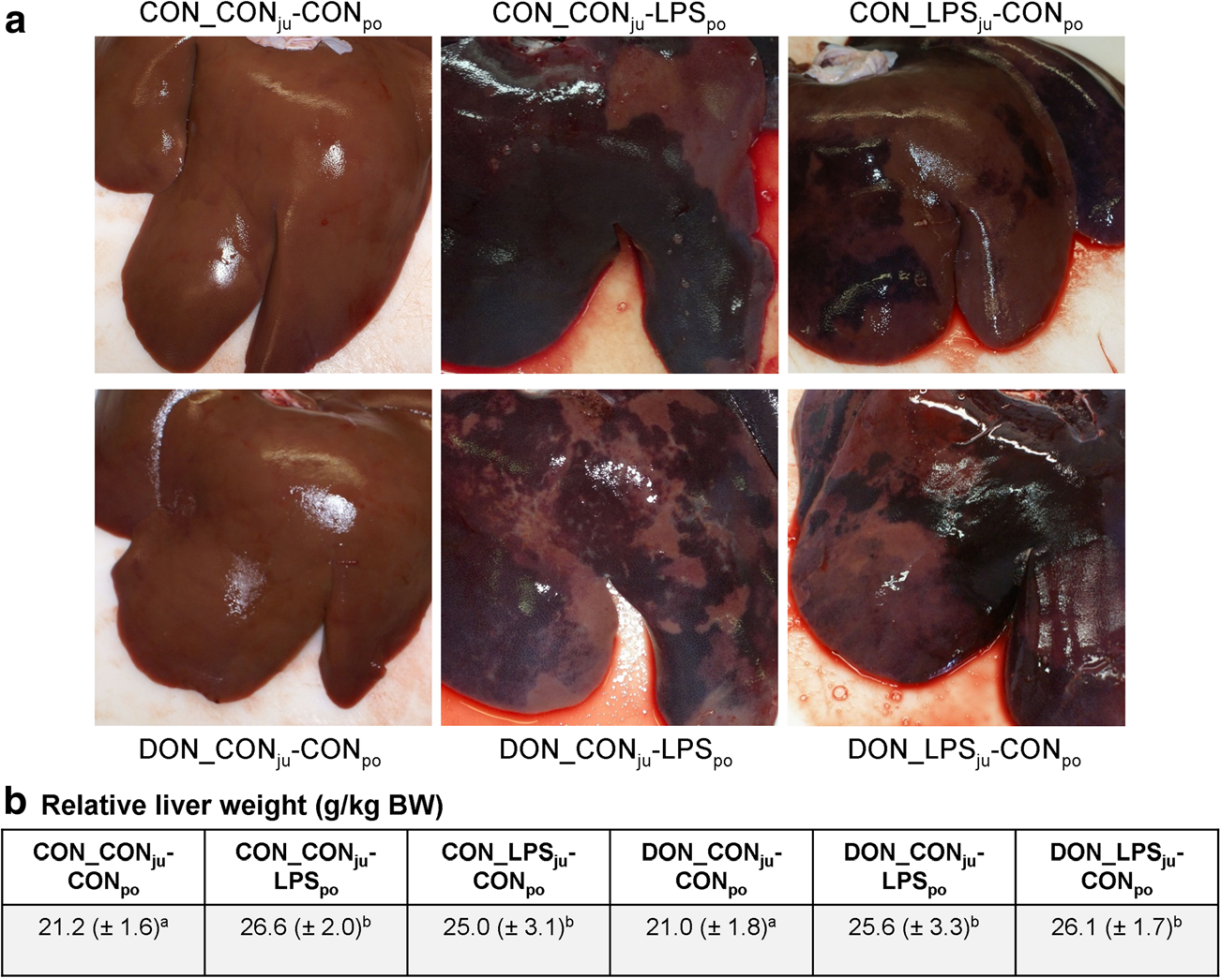
**a** Representative photographs of livers, reflecting the common hepatic macroscopy in each experimental group. Haemorrhages are visible in all LPS-treated groups, but not in groups with saline infusion, irrespective of dietary treatment. **b** Relative liver weight (g/kg BW) in LPS-treated groups was significantly higher compared to saline-infused counterparts, irrespective of diet. Values are presented as LSMeans (±SEM), and those with uncommon *superscripts* are significantly different from each other (*p* < 0.05)

**Fig. 3:**
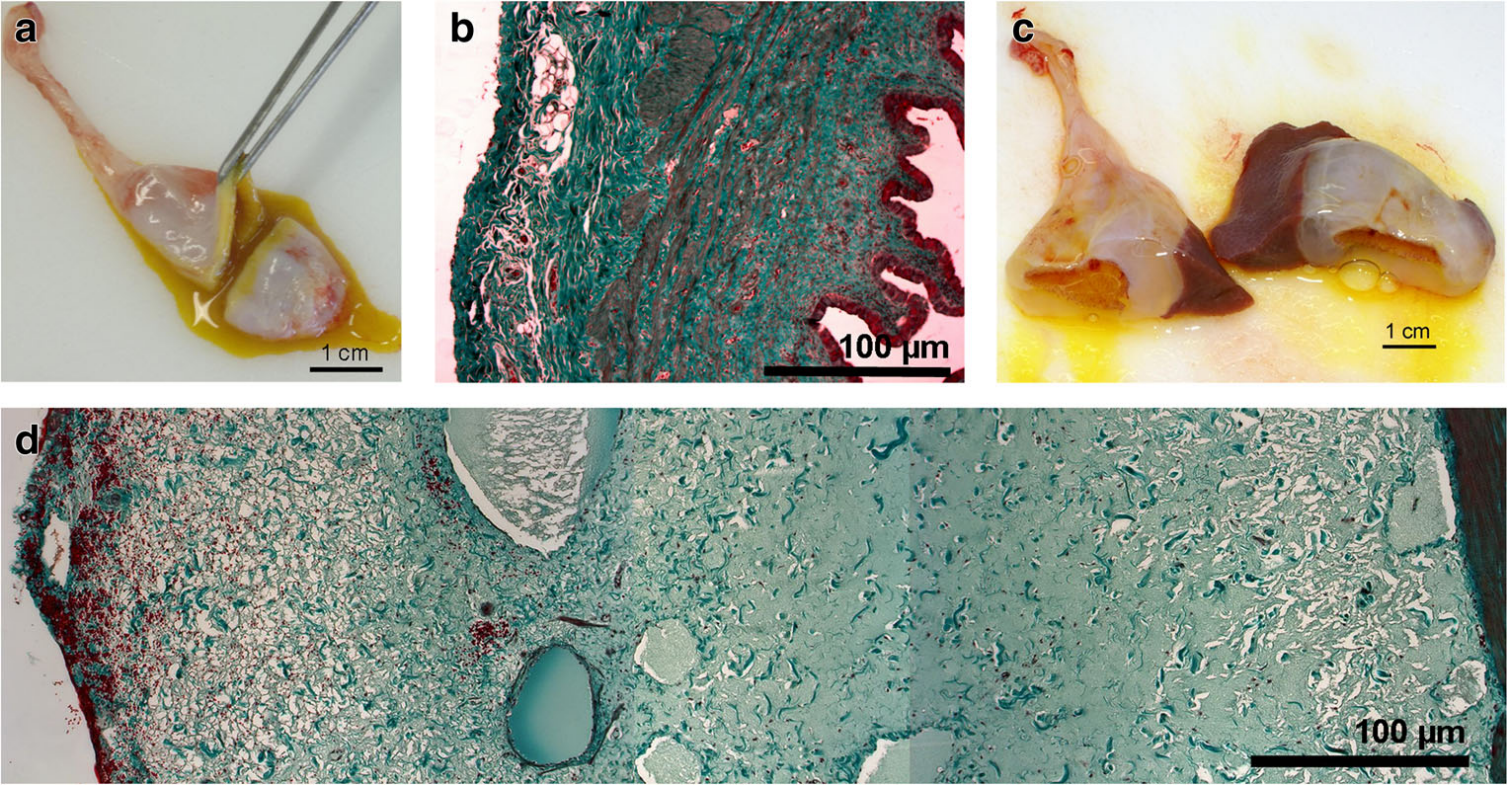
Representative macroscopy and microscopy of gallbladders. **a** Macroscopic appearance of gallbladder in control animals with physiological wall thickness. **b** Histological appearance of gallbladder wall in control animals (Masson-Goldner trichrome staining). Epithelium is stained in dark red (*right side*), myocytes in light red and connective tissue in turquoise. Serosa is visible at the left side of specimen. **c** Macroscopic appearance of gallbladder in LPS-treated animals with visible thickining of gallbladder wall. **d** Histological appearance (Masson-Goldner trichrome staining) of gallbladder wall in LPS-treated animals. Subserosa appears markedly enlarged and edematous, muscularis (red structure) just visible on the *right side*

Animals treated with LPS showed macroscopically visible alterations in the liver (Fig. 2a), in particular haemorrhages, mainly comprising petechiae, ecchymoses and sugillations on the livers surface. Accordingly, an increase in relative liver weight was observed in all LPS challenged groups (Fig. 2b), irrespective of dietary treatment or infusion site. Another striking observation was a marked thickening of the gallbladder wall in LPS-treated pigs (Fig. 3c). The texture of the wall appeared to be gelatinous, and the altered wall reduced visibly the gallbladder lumen compared to saline-infused groups (Fig. 3a). Exemplary histology (Fig. 3d) of LPS-infused specimen revealed a subserosal oedema as cause for the thickened gallbladder wall in contrast to saline-infused specimens (Fig. 3b).

### Histopathology of the liver

**Fig. 4:**
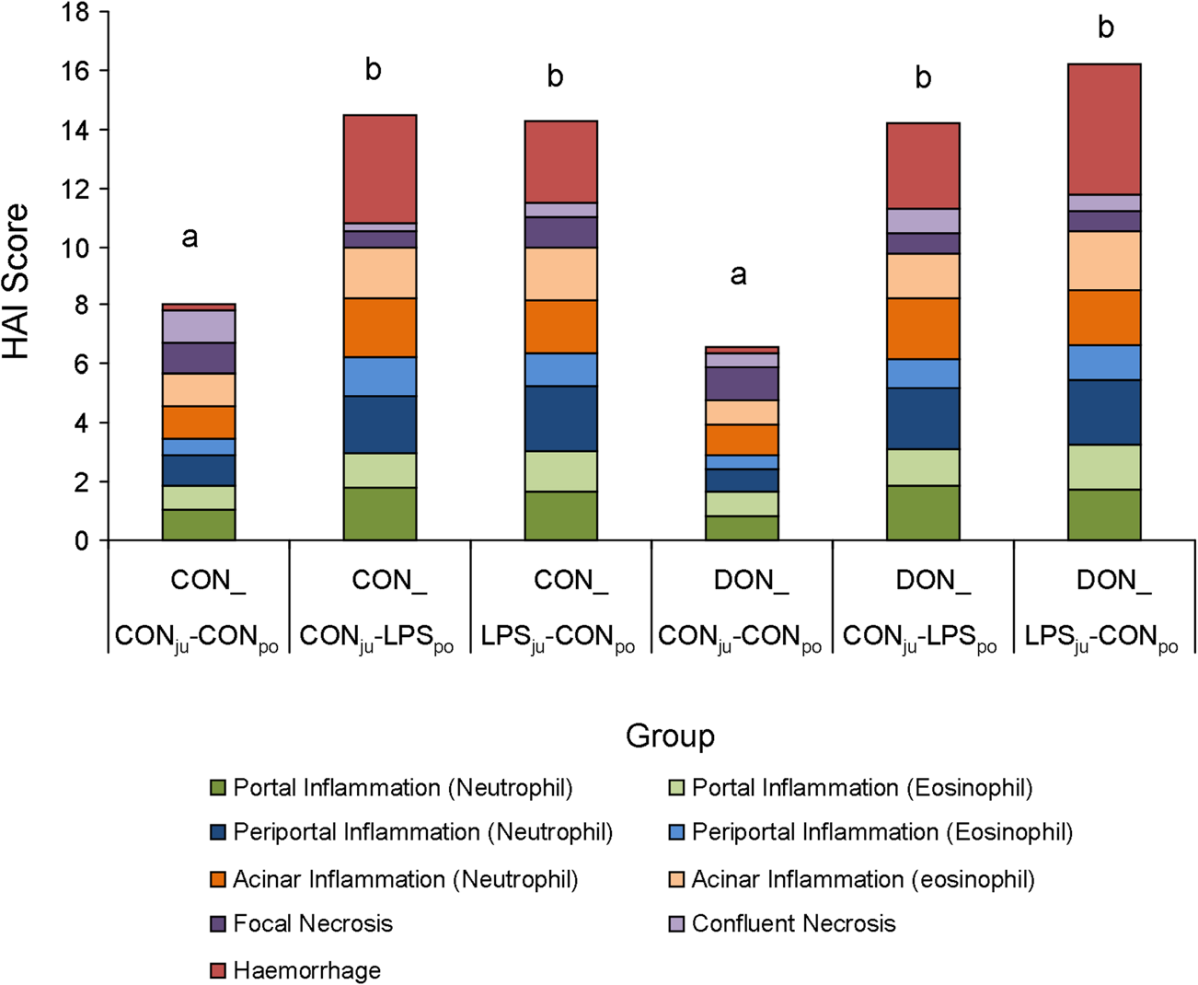
Histology activity index (*HAI*) of the liver. Pigs were fed a control (*CON*) or DON-contaminated diet and infused with LPS or saline (CON) into jugular (*ju*) or *portal* (*po*) region; columns with unlike *superscripts* differ significantly from each other (Kruskal-Wallis with Dunn’s post-hoc test, *p* < 0.05)

Inflammation, haemorrhage and necrosis were evaluated and scored in HE stained liver sections, Fig. S1 providing examples of each histopathological alteration. Scores of individual parameters were summed up for a cumulative HAI in order to gain a global measure of histopathological damage. Chronic dietary DON exposure alone did not affect the liver of pigs compared to those fed control feed (saline-infused groups), whereas administration of LPS increased the cumulative HAI, irrespective of dietary treatment or infusion site (Fig. 4). This effect was seen in all LPS-treated groups with the numerically highest score in DON_LPS_ju_-CON_po_ group. The increased cumulative HAI could be mainly ascribed to haemorrhage and inflammation (Fig. 4, Table 1). Increased infiltration of inflammatory cells, especially neutrophil granulocytes, was found in all parts of liver lobules (portal, periportal and acinar area) after LPS infusion. Infiltration of eosinophil granulocytes was less pronounced in response to LPS (Table 1). Necrotic lesions were observed to a very low degree in all experimental groups with no impact of treatment.

**Table 1:** Effect of DON feeding and pre- or post-hepatic LPS infusion on liver histopathology

Histopathological parameter	CON CON_ju_-CON_po_	CON CON_ju_-LPS_po_	CON LPS_ju_-CON_po_	DON CON_ju_-CON_po_	DON CON_ju_-LPS_po_	DON LPS_ju_-CON_po_	*p* value
Portal (total)	1.9 (±0.4) a	3.0 (±0.9) b	3.1 (±0.6) b	1.6 (±0.4) a	3.1 (±0.6) b	3.3 (±0.7) b	<0.001
Neutrophil	1.1 (±0.2) a	1.8 (±0.8) b	1.7 (±0.4) b	0.9 (±0.2) a	1.9 (±0.6) b	1.8 (±0.5) b	0.001
Eosinophil	0.8 (±0.4) a	1.2 (±0.8) ab	1.4 (±0.5) b	0.8 (±0.4) a	1.3 (±0.3) b	1.5 (±0.3) b	0.021
Periportal (total)	1.6 (±0.6) a	3.3 (±0.9) b	3.3 (±0.6) b	1.3 (±0.4) a	3.0 (±0.7) b	3.4 (±0.9) b	<0.001
Neutrophil	1.1 (±0.5) a	1.9 (±0.2) b	2.2 (±0.3) b	0.8 (±0.3) a	2.1 (±0.2) b	2.3 (±0.4) b	<0.001
Eosinophil	0.5 (±0.2) a	1.3 (±0.9) b	1.1 (±0.4) b	0.5 (±0.4) a	0.9 (±0.5) ab	1.2 (±0.9) ab	0.033
Acinar (total)	2.3 (±0.5) a	3.8 (±0.9) b	3.6 (±0.9) b	1.9 (±0.7) a	3.6 (±0.8) b	3.8 (±1.1) b	<0.001
Neutrophil	1.1 (±0.2) a	2.0 (±0.6) b	1.8 (±0.5) b	1.0 (±0.0) a	2.1 (±0.2) b	1.8 (±0.4) b	<0.001
Eosinophil	1.1 (±0.4)	1.8 (±0.9)	1.8 (±0.8)	0.9 (±0.7)	1.6 (±0.8)	2.0 (±0.8)	0.065
Neutrophil (total)	3.3 (±0.5) a	5.8 (±1.4) b	5.7 (±0.9) b	2.6 (±0.4) a	6.0 (±0.8) b	5.8 (±1.1) b	<0.001
Eosinophil (total)	2.4 (±0.7) ab	4.3 (±2.3) bc	4.3 (±1.1) c	2.1 (±1.1) a	3.8 (±1.4) bc	4.7 (±2.0) c	0.008
Inflammation (total)	5.7 (±0.9) a	10.0 (±2.4) b	10.0 (±1.5) b	4.8 (±1.0) a	9.8 (±1.6) b	10.5 (±2.5) b	<0.001
Confluent necrosis	1.1 (±0.8)	0.3 (±0.5)	0.5 (±0.9)	0.5 (±0.8)	0.9 (±0.8)	0.5 (±0.8)	0.35
Focal necrosis	1.0 (±0.3)	0.5 (±0.5)	1.0 (±1.0)	1.1 (±0.5)	0.6 (±0.4)	0.8 (±0.4)	0.29
Haemorrhage	0.3 (±0.4) a	3.7 (±1.9) b	2.8 (±1.7) b	0.2 (±0.4) a	2.9 (±1.9) b	4.4 (±0.6) b	<0.001
Cumulative HAI	8.1 (±1.88) a	14.5 (±3.08) b	14.3 (±2.35) b	6.6 (±1.46) a	14.2 (±3.78) b	16.2 (±3.08) b	<0.001

Individual histological parameters, the sum of different parameters regarding inflammation and the cumulative histology activity index (HAI) are presented as means (±SD). The *p* value of the fixed factor “group” is also provided. Different lowercase letters in a row indicate significance between groups, *p* < 0.05 (Dunn’s post-hoc test)

*CON* control feed, *DON* DON feed, *CON*_*ju*_ saline infusion jugular catheter, *LPS*_*ju*_ LPS infusion jugular catheter, *CON*_*po*_ saline infusion portal catheter, *LPS*_*po*_ infusion portal catheter

### Clinical chemical parameters

All measured parameters were evaluated 30 min prior to infusion (base level), and because at this time no infusion regimen was implemented yet, only diet could have an impact. However, no differences between groups were detected, and thus, feeding a DON-contaminated diet for 4 weeks showed no direct effect on clinical chemistry. Regarding both placebo-infused groups (CON_CON_ju_-CON_po_ vs. DON_CON_ju_-CON_po_) during the entire experimental period, no differences in clinical chemical parameters were detectable between both groups.

#### Liver enzymes

The enzymes AST, γGT and ALP (Table 2) were tested as markers for hepatocyte integrity and cholestasis and all parameters showed a significant three-way interaction between the main factors. AST levels ranged all below the upper physiological limit until 120 min *post infusionem*. The activity increased at 180 min after infusion start and exceeded the upper limit in all LPS infused groups, whereas NaCl-infused groups showed no pathological findings. Furthermore, LPS challenge significantly increased γGT level until 180 min without exceeding the physiological upper limit, whereas γGT of both control-infused groups (CON_CON_ju_-CON_po_ and DON_CON_ju_-CON_po_) decreased slightly in time (*p* = 0.08). In control-fed pigs, there was an impact of infusion site at 180 min: jugular (post-hepatic) LPS-infusion yielded significantly higher γGT levels compared to the portal-infused counterpart (*p* ≤ 0.01) in portal blood samples. In DON-fed groups, this site effect was not detectable.

**Table 2:** Effect of dietary mycotoxin and subsequent immune challenge on clinical chemical parameters (LSMeans)

	Group						*p* values			
	CON_ CON_ju_-CON_po_	CON_ CON_ju_-LPS_po_	CON_ LPS_ju_-CON_po_	DON_ CON_ju_-CON_po_	DON_ CON_ju_-LPS_po_	DON_ LPS_ju_-CON_po_	Group	Site^a^	Time	g*s*t
Time	AST (<35 IU/L)									
−30	32.2	33.0	32.6	32.1	32.5	31.0	0.18	0.97	<0.001	0.03
60	31.8	30.5	31.5	33.1	32.9	29.7				
120	28.7	31.9	31.3	31.1	30.6	31.9				
180	28.0	39.2	38.2	33.2	38.1	39.1				
PSEM	1.1									
	γGT (<45 IU/L)									
−30	30.6	30.6	30.6	30.3	30.9	30.3	<0.001	0.36	0.002	0.002
60	30.0	33.2	33.4	27.5	36.9	35.1				
120	29.0	31.9	36.8	27.9	35.0	36.2				
180	27.4	31.7	38.5	26.6	34.5	32.9				
PSEM	0.9									
	ALP (<170 IU/L)									
−30	134.2	134.3	132.0	129.7	134.6	132.7	0.01	0.93	<0.001	0.03
60	135.3	141.4	146.8	135.2	143.2	160.2				
120	132.7	140.6	135.7	124.5	122.4	152.6				
180	110.0	174.4	180.1	122.0	146.0	236.7				
PSEM	8.4									
	ALB (18–31 g/L)									
−30	37.8	38.0	38.0	37.5	37.8	37.7	0.28	0.39	<0.001	0.26
60	35.9	36.4	37.3	35.6	37.0	35.3				
120	35.3	33.7	34.6	35.6	33.0	33.5				
180	34.2	32.5	34.6	35.1	31.6	31.3				
PSEM	0.6									
	Total protein (55–86 g/L)									
−30	52.9	53.1	53.2	52.7	53.0	53.0	0.03	0.50	<0.001	<0.001
60	50.0	50.8	52.1	49.6	51.2	48.7				
120	49.6	45.4	47.8	48.4	44.7	45.3				
180	48.6	43.9	46.5	48.9	44.2	42.1				
PSEM	0.701									

Physiological reference values are provided in brackets (Kraft and Dürr 2014)

*AST* aspartate aminotransferase (IU/L), *γGT* gamma glutamyl transferase (IU/L), *ALP* alkaline phosphatase (IU/L), *ALB* albumin (g/L), total protein (g/L), *CON* control feed, *DON* DON feed, *CON*_*ju*_ saline infusion jugular catheter, *LPS*_*ju*_ LPS infusion jugular catheter, *CON*_*po*_ saline infusion portal catheter, *LPS*_*po*_ portal infusion portal catheter, *PSEM* pooled standard error of the mean

^a^As there was no difference between sampling sites (*V. jugularis ext.* and *V. portae hepatis*), mean value of jugular and portal samples was calculated and is shown in the table

ALP levels increased until 180 min in response to LPS-infusion and were significantly higher compared to saline-infused groups. They also exceeded the upper physiological limit of 170 IU/L slightly in group CON_CON_ju_-LPS_po_ and CON_LPS_ju_-CON_po_ 180 min *post infusionem*, but in group DON_LPS_ju_-CON_po_, this increase was much stronger at the same time. In control-fed groups, the ALP response to LPS was fairly homogenous, with no difference between infusion sites. However, in DON-fed groups, infusion site of LPS had a dramatic impact: jugular-infused pigs showed much higher ALP values at 180 min as their portal-infused counterparts (236.7 vs. 146.0 IU/L; *p* < 0.05), whereby the latter did not even exceed the upper physiological level. This strong impact was also reflected in a significant difference between the DON_LPS_ju_-CON_po_ and the control-fed equivalent CON_LPS_ju_-CON_po_ (p_ju_ = 0.008; p_po_ = 0.09).

GLDH activity was below the limit of detection (LOD) in all samples.

#### Albumin and total protein

The protein synthesis capacity of the liver was monitored by detection of serum albumin (liver-specific) and total serum protein. Base level albumin concentration was slightly higher than the reference value given for pigs (Kraft and Dürr 2014) and significantly decreased in the course of the observation period in all groups (Table 2). In contrast, total protein contents were slightly lower than the reference value (Kraft and Dürr 2014), but decreased in time similar to albumin concentration. However, this decrease was more pronounced in LPS-infused groups compared to saline-infusion as evidenced by a significant three-way interaction. Thus, at 180 min, saline-infused groups, irrespective of diet, showed significantly higher protein values compared to LPS-infused groups. Moreover, in jugular-infused LPS group, diet had also a significant effect, with DON-fed pigs showing a lower protein concentration than the respective control-fed group in portal (pre-hepatic) blood (*p* < 0.001), but not in jugular-drawn samples.

#### Total bilirubin

**Fig. 5:**
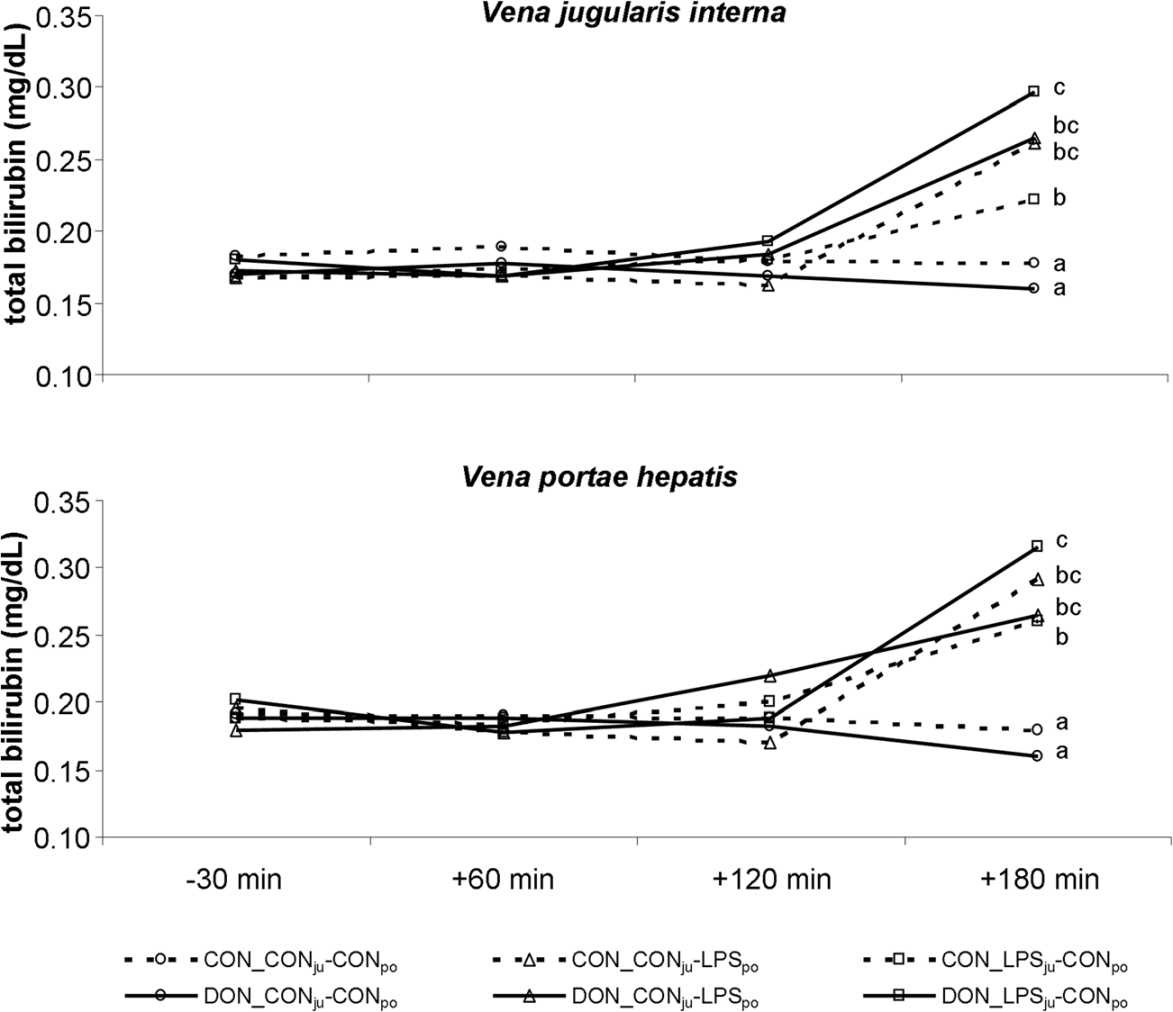
Bilirubin concentrations in peripheral and portal blood. Effect of chronic enteral *Fusarium* toxin deoxynivalenol (*DON*) exposure and pre- or post-hepatic *E. coli* LPS infusion on total bilirubin concentrations in *V. jugularis interna* and portal *V. portae hepatis*. Bilirubin was significantly increased in LPS groups 180 min after start of infusion compared to placebo-infused groups. Additionally, *DON_LPS*_*ju*_ *-CON*_*po*_ had significantly higher concentrations compared to their control-fed counterparts *CON_LPS*_*ju*_ *-CON*_*po*_, whereas this was not the case for portal-infused LPS groups. Reference value of total bilirubin (Kraft and Dürr 2014): ≤0.25 mg/dL in blood. Main effects (*F* test) were calculated at group (g) p_group_ = 0.008; infusion site (s) p_site_ = 0.371; time (*t*) p_time_ < 0.001. Interaction p_g × s × *t*_ < 0.001; *different letters* indicate significant differences between groups (*p* < 0.05, post-hoc *t* test)

The concentration of total bilirubin (Fig. 5) increased 180 min *post infusionem* in all LPS-infused groups and exceeded the physiological upper limit of 0.25 mg/dL (Kraft and Dürr 2014), except for CON_LPS_ju_-CON_po_ in jugular blood samples. The bilirubin levels remained constantly low throughout the entire experimental period in saline-infused groups and were significantly lower compared to LPS groups at 180 min. Moreover, there was a strong impact of diet in jugular-infused LPS groups, with DON_LPS_ju_-CON_po_ showing significantly higher values compared to CON_LPS_ju_-CON_po_ (*p* < 0.05). This difference in dietary impact was not detectable in portal-infused groups.

### Impact of DON on mitochondrial function

**Fig. 6:**
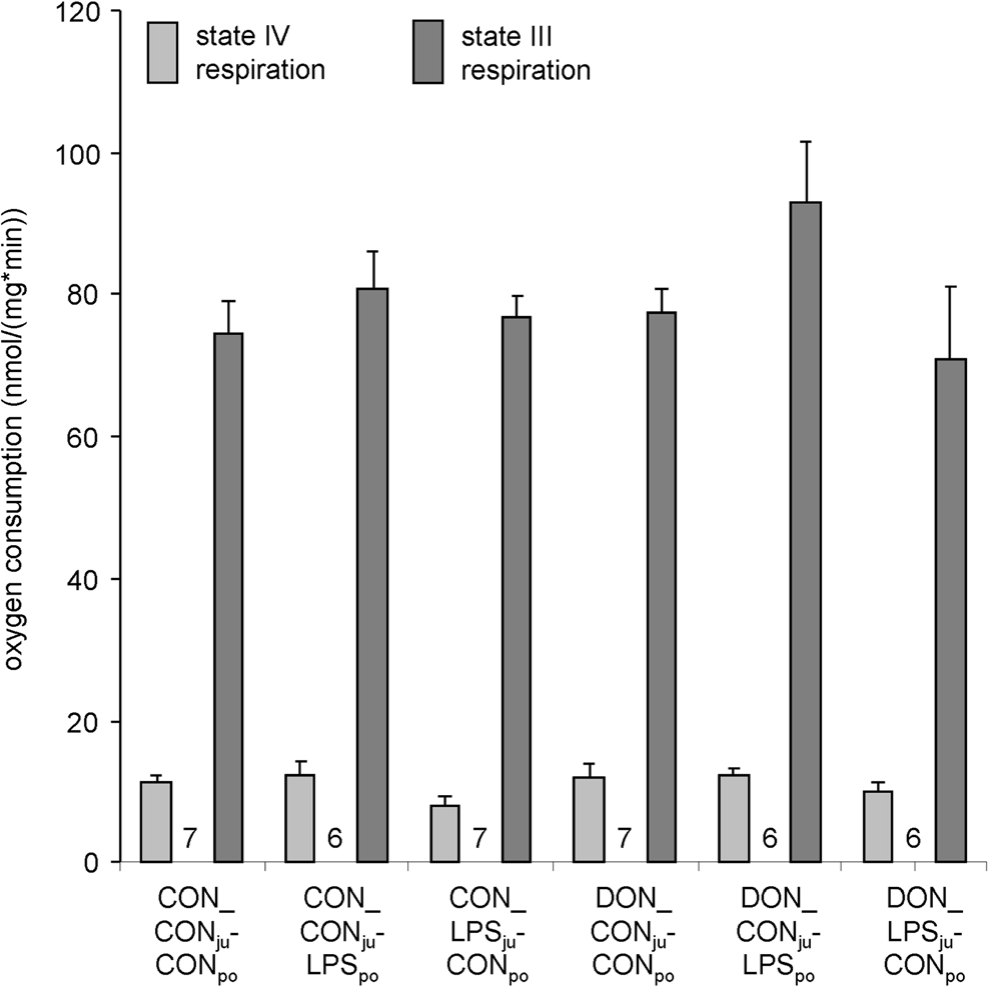
Effect of DON and subsequent LPS-infusion on states III and IV respirations of porcine liver mitochondria. Liver mitochondria were isolated from animals fed with control (CON)- or DON (DON)-contaminated diet for 4 weeks and subsequently infused for 1 h with either LPS (7.5 μg/kg BW) via a jugular or portal catheter (LPS_ju_ or LPS_po_) or NaCl after sacrifice. In isolated liver, respiration was estimated using malate/glutamate as substrates in presence (state III) and absence (state IV) of ADP. Mean values of state III and state IV respiration of 6–7 animals per group (*n* between *bars*) were analysed by ANOVA. No statistically significant differences were found between groups ± SEM

Liver mitochondria of pigs receiving DON-contaminated feed for 4 weeks were compared with those of the control feed group. As shown in Fig. 6, the measured state IV and state III respirations were not significantly different between CON_CON_ju_-CON_po_ and DON_CON_ju_-CON_po_. The acute challenge either by jugular or by portal LPS application had no significant impact on mitochondrial respiration independent of the previous feeding regime.

Mitochondrial preparations were subsequently used to assess the CRC in presence or absence of the inhibitor of PTP CsA. Thus, mitochondria were not sensitized to undergo the permeability transition. Since enhanced CRC, this parameter was estimated in buffer supplemented with 1 or 10 mM PO_4_^3−^ (P_i_), respectively. Measured CRC data were in the presence of CsA 8 to 10 times higher. Neither DON feeding nor acute application of LPS significantly changed the CRC in absence of CsA or P_i_ setting (Table 3).

**Table 3:** Effect of DON-contaminated feed and a subsequent LPS challenge on Ca^2+^ retention capacity (CRC) of porcine liver mitochondria

Accumulated Ca^2+^ (nmol/mg mitochondrial protein)	CON_ CON_ju_-CON_po_	CON_ CON_ju_LPS_po_	CON_ LPS_ju_-CON_po_	DON_ CON_ju_-CON_po_	DON_ CON_ju_-LPS_po_	DON_ LPS_ju_-CON_po_
1 mM P_i_	130 ± 22	113 ± 13	133 ± 15	105 ± 14	129 ± 16	126 ± 10
1 mM P_i_ + CsA	908 ± 56	850 ± 58	964 ± 67	817 ± 42	921 ± 44	870 ± 68
10 mM P_i_	90 ± 11	70 ± 9	94 ± 20	75 ± 13	83 ± 11	88 ± 18
10 mM P_i_ + CsA	850 ± 44	792 ± 90	829 ± 66	783 ± 54	833 ± 55	870 ± 78

Mean values (±SEM) of each experimental setup (1, 10 mM P_i_, with or without CsA) of 6–7 animals per group were analysed by ANOVA. No statistically significant differences were found between experimental groups

*CON* control feed, *DON* DON feed, *CON*_*ju*_ saline infusion jugular catheter, *LPS*_*ju*_ LPS infusion jugular catheter, *CON*_*po*_ saline infusion portal catheter, *LPS*_*po*_ portal infusion portal catheter, *P*_*i*_ inorganic phosphate, *CsA* cyclosporine A

## Discussion

The aim of our study was to investigate whether the functional hepatic capacity is modified by a chronic dietary DON exposure and results in an attenuated response of liver mitochondria and function to an immune challenge in vivo.

### Liver morphology

We could not detect a detrimental impact of dietary DON alone on hepatic morphology, neither macroscopically nor microscopically. This is in accordance with previous studies in young pigs (Stanek et al. 2012) and pregnant sows (Tiemann et al. 2008), applying oral DON concentrations in the same range as in our present study. In contrast, Gerez et al. (2015) observed histological lesions of the liver described as disorganization of hepatic cords, cytoplasmic vacuolization of hepatocytes and focal necrosis after feeding a diet contaminated with DON (1.5 mg/kg feed) or a combination of DON, nivalenol and zearalenone to 5-week-old male piglets. This could be attributed to the age of piglets investigated, being only 5 weeks old whereas the animals in our experiments were 14 weeks at the end of trial, suggesting a higher susceptibility to DON in younger developing animals.

Severe haemorrhages of the liver were observed after LPS administration, also reflected in the greater relative liver weight as reported previously in dogs (MacLean et al. 1956), broilers (Mireles et al. 2005) and pigs (Stanek et al. 2012). The histopathological examination of liver samples in LPS-infused pigs confirmed the haemorrhage and showed also a marked infiltration of granulocytes in all regions of liver lobules. This immigration of leukocytes into the liver tissue started already 15 min after start of infusion as visible in the drop of leukocyte differential counts (Tesch et al. 2015) and persisted until the final sampling at 180 min. The early shift from blood stream to liver was most pronounced in neutrophils, emphasizing their importance as part of the innate immune system in eliminating bacterial agents such as LPS. The lack of LPS-induced hepatic necrosis, often triggered by infiltration of neutrophils causing oxidative stress (Ramaiah and Jaeschke 2007) and reported already in endotoxaemic models (Li et al. 2012; Depboylu et al. 2013), might be explained by the rather short observation period in our study (3 h). However, no additive, synergistic or antagonistic impact of DON feeding on the LPS-induced morphological hepatic changes was detectable in our study, in contrast to earlier investigations reporting a more attenuating impact of dietary DON (Stanek et al. 2012).

Additionally, gallbladder wall showed severe subserosal oedema after LPS administration, but not in response to chronic DON feeding. In vitro, canine epithelial gallbladder cells showed increased mucin secretion and increased expression of cyclooxygenase-2 and prostaglandin E_2_ in response to 100 μm/mL LPS after 8 to 24 h of incubation (Kim et al. 2003a). Patients, suffering from acute hepatitis, showed gallbladder wall thickening, mainly ascribed to swelling of the serosal and muscular layer, but not the mucosa (Kim et al. 2003b). The authors conclude that the reasons for gallbladder wall thickening are inflammatory events in response to necrosis and inflammation of the liver. Cullen and co-workers (2000) reported pathohistological alterations in gallbladder walls of opossums, challenged with *E. coli* LPS in a dose-response trial, but with even more severe responses such as haemorrhages, necrosis and mucosal sloughing using comparable dosages (5 μg LPS/kg BW).

### Liver and mitochondrial function

LPS-infusion as such increased functional parameters such as AST, γGT, ALP and total bilirubin, albeit not always severely. In contrast to hepatic morphology, we did observe a strong interaction of diet and LPS infusion site, in particular for ALP and bilirubin. Both parameters were significantly higher in DON-fed, jugular LPS-infused pigs, compared not only to NaCl-infused animals but also to their CON-fed, LPS-infused counterparts. Interestingly, this interaction depended entirely on the LPS route of entry: jugular (= post-hepatic) route revealed this dietary modulation, whereas portal (=pre-hepatic) infusion did not show such dietary discrimination.

Hyperbilirubinemia, a key sign of hepatic dysfunction, can be caused by three pathogenic forms: haemolysis (pre-hepatic), intra-hepatic dysfunction and post-hepatic cholestasis (Chand and Sanyal 2007). Haemolysis as a cause for hyperbilirubinemia can be excluded in our study, because no LPS or DON effect on red blood cell counts and indices was found in these pigs as reported earlier (Bannert et al. 2015). Another possible explanation could be cholestasis, due to either intra-hepatic or post-hepatic alterations. Two parameters, γGT and ALP (Poupon 2015) are common indicators for an occurring cholestasis (Kraft and Dürr 2014) and both were generally increased by LPS, albeit in the case of γGT not yet above the upper physiological range. Additionally, both showed a strong interaction of diet and LPS entry site. The most common reason for post-hepatic cholestasis is cholecystitis, accompanied with gallstones in 90–95% of cases (Runner et al. 2014), resulting also in gallbladder wall thickening. Gallstones were not detected at all after sacrifice of animals, excluding this reason for cholestasis and the detected gallbladder wall thickening. In our study, the gallbladder wall showed a subserosal oedema likely due to inflammatory events in the liver as was already reported for patients with acute hepatitis (Kim et al. 2003b). Thus, hyperbilirubinaemia should have occurred due to intra-hepatic events. One such mechanism reported to increase bilirubin (total, conjugated and unconjugated bilirubin) is the downregulation of UDP-glucuronyltransferase 1A1 (UGT1A1) in hepatocytes via an NFκB-dependent pathway in response to LPS (Shiu et al. 2013). UGT1A1 is an enzyme involved in conjugation not only of various substances such as bilirubin (Tukey and Strassburg 2000) but also of xenobiotics such as deoxynivalenol (Maul et al. 2015), and therefore might be at least partially responsible for increased concentrations of (unconjugated) bilirubin and the dietary modulation observed. Indeed, UGT1A1 mRNA expression was significantly downregulated in LPS-treated groups (unpublished data), but with no apparent discrimination between LPS-infusion site and dietary treatment. However, this might be differentially expressed on a protein basis or activity of the enzyme, contributing to the observed detrimental effect of DON.

The ADP-dependent respiration is a sensitive tool for unravelling harmful effects of potentially toxic contaminations. This is why a variety of proteins and enzymatic systems, such as the respiratory chain, the citric acid cycle and the F_1_F_O_-ATPsynthase, contribute to the state III respiration (phosphorylating respiration) and thus to the mitochondrial energy metabolism.

In vivo measurements showed that DON concentrations were up to 14 ng/mL in the blood stream and up to 1300 ng/g in chyme (Dänicke et al. 2004). Permeability transition pore was reported in isolated mitochondria for T-2 toxin and *Fusarium* toxin zearalenone, at concentrations of 7.5 nM (T-2) and 30 μM (zearalenone) (Bouaziz et al. 2006). Severe treatment of rats with a T-2 mycotoxin (0.5 mg/kg for 10 h) affected the respiration of liver mitochondria (Pace 1983). Mycotoxins could affect the mitochondrial function by various ways. Besides direct effects on respiration, DON may also affect the mitochondrial protein configuration as shown for trichothecin (Tcin) in yeast mitochondria (Bin-Umer et al. 2011). It is conceivable that the porcine mitochondria protein configuration has changed in response to 29 days of DON exposure; however, we found no indication for this mechanism in our approach. The data on porcine liver mitochondria indicate that DON feeding over a 4-week period did not modify the functional parameters investigated. Despite massive morphological effects as reflected by HAI score, the mitochondrial function was also not affected by LPS challenge. These results are in line with the absence of GLDH in serum (below LOD), which should increase in case of mitochondrial damage.

## Conclusion

The aim of our study was to investigate whether the functional hepatic capacity is modified by dietary DON exposure resulting in an attenuated response of liver mitochondria and function to an immune challenge in vivo. DON showed a clear priming effect on liver function (ALP, bilirubin), but in contrast to our initial hypothesis, rather aggravated the subsequent LPS response than alleviating it. However, this priming was not reflected in hepatic morphology.

ALB, albumin; ALP, alkaline phosphatase; ANOVA, analysis of variance; APR, acute phase reaction; AST, aspartate aminotransferase; BW, body weight; CaG5N, calcium green-5N; CON, control feed; CON_ju_, saline infusion jugular catheter; CON_po_, saline infusion portal catheter; CRC, calcium retention capacity; CsA, cyclosporine A; DM, dry matter; DON, deoxynivalenol feed; GLDH, glutamate dehydrogenase; HAI, histology activity index; HE, haematoxylin and eosin; IL, interleukin; i.p., intra-peritoneal; ju, jugular catheter; LBP, LPS-binding protein; LPS, lipopolysaccharides; LPS_ju_, LPS infusion jugular catheter; LPS_po_, LPS infusion portal catheter; LSMeans, least square means; po, portal catheter; PSEM, pooled standard error of means; PTP, permeability transition pore; RCI, respiratory control index; TLR4, toll-like receptor 4; TNF-α, tumour necrosis factor α; UGT1A1, UDP-glucuronyltransferase 1A1; γGT, gamma glutamyl transferase

## Electronic supplementary material

ESM 1 (PPTX 5612 kb)
