# Supplementary material for: Chronic DON exposure and acute LPS challenge: effects on porcine liver morphology and function
Source: Mycotoxin Res. 2017 May 4;33(3):207–18. doi: 10.1007/s12550-017-0279-9 (PMC5511606; doi:10.1007/s12550-017-0279-9)
Supplement: Supplementary file 1 — (PPTX 5612 kb) [file 12550_2017_279_MOESM1_ESM.pptx]

## Slide 1
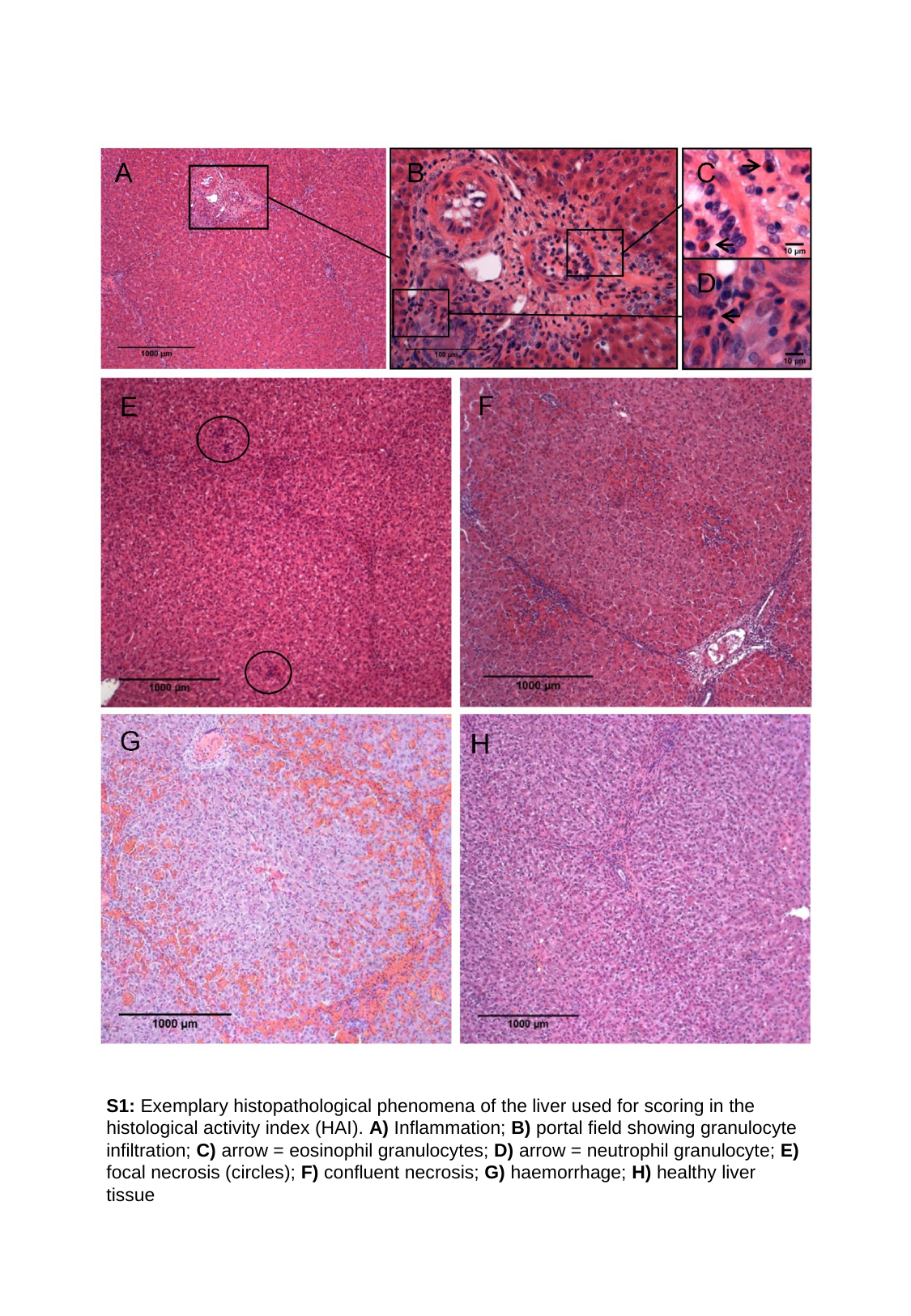

S1: Exemplary histopathological phenomena of the liver used for scoring in the histological activity index (HAI). A) Inflammation; B) portal field showing granulocyte infiltration; C) arrow = eosinophil granulocytes; D) arrow = neutrophil granulocyte; E) focal necrosis (circles); F) confluent necrosis; G) haemorrhage; H) healthy liver tissue
